# Implementing policies and predictive stochastic models to attend to borderline personality disorder crises: rationalising ssri antidepressants prescription in suicide prevention

**DOI:** 10.1192/j.eurpsy.2024.1633

**Published:** 2024-08-27

**Authors:** C. G. Lazzari

**Affiliations:** Community Mental Health, UK NHS, BRIGHTON, United Kingdom

## Abstract

**Introduction:**

We are facing increased suicide attempts and deliberate self-harm from persons with borderline personality disorder (BPD) who are also on antidepressants, multiple antidepressant prescriptions and antidepressant augmentations. Our previous observations suggest that antidepressants might increase suicide attempts in those on this medication and who have BPD. The absent response to antidepressants is due mainly to the comorbid dysthymia, cyclothymia, rumination, autism and ADHD in BPD.

**Objectives:**

To generate forecasting models and preventive policies to deal with BPD crises and improve the effectiveness of the UK National Healthcare Service (NHS) in suicide prevention.

**Methods:**

The underlying analysis framework is stochastic forecasting. We used current knowledge and data to complete systematic future predictions extracted from recent trends. A logical-mathematical model generated the required expressions. The software for logic prediction and annotation was Wolfram Alpha (Wolframalpha.com). The four parameters for stochastic predictions are, BPD (A), antidepressant No. 1 (B), antidepressant No. 2 (C), and suicide attempts (D). Boolean function metrics can help analyse the impact and truth of forecast modelling with truth density.

**Results:**

The logic expression for suicide prediction due to liberal antidepressant prescribing is Ψ = A intersects B, intersects C, intersects D; that is,. Ψ = A ∩ B ∩ C ∩ D, which yields a Boolean truth density of 6.25%. The truth table always has a positive outcome as long as any of the factors exist except when none is present.

**Conclusions:**

The predictive Boolean function and truth table suggest that suicide presentation is predictable if there is a prescribing of one or more antidepressants in BPD and if there is an antidepressant augmentation or dose maximisation. We speculate that SSRI antidepressants block self-regulatory mechanisms of fear of death while triggering impulses to self-harm and suicide from overstimulation of SSRI receptors. Without fear mechanisms, death by suicide is felt as not terrifying.

**Disclosure of Interest:**

None Declared

